# A Multilevel Network Peer Intervention Among Student Men Who Have Sex With Men Attending University: Protocol for an Implementation-Effectiveness Before-After Cohort Study

**DOI:** 10.2196/77078

**Published:** 2026-01-23

**Authors:** Jingpei Xu, Zhen Dai, Yushu Qiu, Liang Wang

**Affiliations:** 1 Department of Sexually Transmitted Disease, AIDS Prevention and Control, Chengdu Center for Disease Control and Prevention (Chengdu Institute of Health Supervision), Longxiang Road 4, Wuhou District, Chengdu, Sichuan, 610041, China, 86 15208496150

**Keywords:** HIV, MSM, university students, internet-based intervention, peer education, cohort study, PEP, men who have sex with men, postexposure prophylaxis

## Abstract

**Background:**

The HIV prevalence among student men who have sex with men (MSM) in China is substantially higher than that in the general student population. However, targeted interventions for this vulnerable population remain limited. While digital technologies and peer-led approaches have shown promise in HIV prevention among MSM, their application in university settings is underexplored.

**Objective:**

This study aims to evaluate the implementation and effectiveness of a multilevel, internet-based peer intervention model in reducing HIV and syphilis incidence, improving prevention behaviors, and increasing uptake of HIV-related services among student MSM attending university in Chengdu.

**Methods:**

This prospective before-and-after self-controlled cohort study will evaluate the effectiveness of a 12-month internet-based intervention targeting university student MSM in Chengdu. A total of 484 HIV-negative student MSM among the MSM population will be recruited through WeChat and Blued. Trained student volunteers, supervised by community-based organization staff, will deliver monthly health education, one-on-one counseling, postexposure prophylaxis navigation, and HIV or sexually transmitted infection (STI) self-testing kits via secure online platforms. Participants will complete behavioral surveys and HIV/syphilis/hepatitis C virus/hepatitis B surface antigen self-tests at baseline, 6 months, and 12 months. Primary outcomes included changes in HIV and syphilis incidence rates; secondary outcomes included changes in sexual behaviors, HIV/STI testing frequency, preexposure prophylaxis/postexposure prophylaxis uptake, and knowledge improvement.

**Results:**

As of May 1, 2025, the intervention materials and training program have been finalized, and recruitment has begun. Enrollment of participants started in April 2025. By the end of April, 127 participants had completed baseline surveys and HIV/STI self-tests.

**Conclusions:**

This study will provide empirical evidence on the feasibility and effectiveness of an internet-based peer intervention for student MSM.

## Introduction

National sentinel surveillance data showed a rapid increase in newly reported HIV cases among young students in China from 2010 to 2019. The HIV prevalence among student men who have sex with men (MSM) in China is substantially higher than that in the general student population, with regional comparisons revealing the highest prevalence in the southwest region [1].

As the economic and cultural center of southwest China [2], Chengdu was estimated to have up to 120,000 active MSM in 2018 [3]. In 2021, the estimated number of college MSM registered on social networking platforms in Chengdu reached 8401, accounting for 2.08% of the total male university student population [4]. Although students were not traditionally considered a high-risk population for HIV infection, surveillance data indicate a rapidly increasing proportion of students infected with HIV/AIDS in the 15- to 24-year age group nationwide [5]. Systematic reviews have reported HIV prevalence rates of 3.8% [6] to 4.4% [5] among university student MSM. Risky sexual behaviors are common [7]; moreover, 85% of student MSM in Chengdu reported meeting sexual partners through mobile social networking platforms [4].

To date, research on university student MSM in China has primarily relied on cross-sectional surveys [6], limiting the understanding of factors driving HIV transmission in this population. While intervention trials targeting MSM populations have been conducted, few have specifically focused on student MSM. The HIV prevalence among university student MSM highlights an urgent need for effective, evidence-based public health strategies tailored to this group [5]. Compared to other high-risk populations, prevention efforts targeting university student MSM in China remain insufficient and under-researched [8].

There is growing evidence that online interventions can effectively reduce HIV risk behaviors and promote HIV testing among MSM populations [9,10]. These findings underscore the potential of digital strategies in reducing HIV or sexually transmitted infection (STI) disparities among student MSM. Given this, it is crucial to evaluate the effectiveness of internet-based intervention programs in this population.

In this study, we aim to establish a cohort of university student MSM and implement a comprehensive intervention through social networking platforms. The intervention will include regular behavioral education, HIV testing promotion, service referrals, and postexposure prophylaxis (PEP) subsidies. Previous research has shown that young people are often influenced by their peers and surrounding environments when adopting health behaviors [11,12]. Moreover, based on Social Cognitive Theory, peer educators who share similar identities and experiences can play a vital role in promoting healthy behaviors among student MSM [13].

In recent years, government regulations on community-based organizations (CBOs) serving the MSM population have become increasingly stringent, particularly regarding their direct engagement with student populations. In Chengdu, while the local Centers for Disease Control and Prevention (CDC) and AIDS Prevention Association have recruited university student volunteers for peer education, these efforts have primarily targeted the general student population and have rarely provided comprehensive, MSM-specific interventions.

Therefore, this study proposes an innovative multilevel network peer intervention model, in which the MSM CBO staff will serve as trainers, and student MSM volunteers will act as peer educators. To our knowledge, this will be the first study in China to explore and implement such a targeted intervention model specifically designed for university student MSM.

## Methods

### Ethical Considerations

Participation in this study will be entirely voluntary, and comprehensive measures will be implemented to ensure participant anonymity and data confidentiality. The study protocol has been reviewed and approved by the Ethics Committee of the Chengdu CDC (Approval No. 2024021).

All participants were required to carefully read the electronic informed consent form prior to enrollment, which will provide detailed information about the study purpose, procedures, potential risks, and benefits. Only those who voluntarily select the “Agree” option will be permitted to proceed with the questionnaire, thereby documenting their informed consent. Participants will have the right to withdraw from the study at any time without any negative consequences.

To prevent coercion or power imbalance between peers, volunteers, and participants were matched across different universities; all volunteers must sign a confidentiality agreement; all participation is anonymous and voluntary. We also conducted dedicated confidentiality training during the pre-project orientation.

To support HIV prevention efforts, the study will provide access to evidence-based online health education, individualized counseling services, and free prevention supplies, including condoms, lubricants, and HIV/STI self-testing kits. Additionally, participants will receive modest financial compensation for completing each survey, in recognition of their time and contribution. Participants received RMB 20 (US $2.83) at baseline, RMB 20 (US $2.83) at the 6-month follow-up, and RMB 20 (US $2.83) at the final 12-month follow-up.

Potential risks primarily will involve the possible inadvertent disclosure of sensitive information related to sexual behavior. However, robust data security and privacy protection protocols will be established, including data encryption, pseudonymization of identifiers, and restricted access to sensitive information, to minimize this risk.

The anticipated benefits of participation—including increased HIV-related knowledge, improved access to prevention services, and facilitation of early detection and timely linkage to care—are expected to outweigh the minimal risks involved.

Findings from this study will be disseminated to local and national public health authorities to inform HIV prevention strategies targeting students belonging to the student MSM population. In addition, study results will be shared with the broader scientific community through peer-reviewed publications and academic conference presentations.

### Study Aims

This prospective cohort study aims to evaluate the effectiveness of a comprehensive Internet-based intervention targeting student MSM attending university. Specifically, it seeks to assess the intervention’s impact on reducing HIV-related high-risk behaviors and lowering the prevalence of HIV and other STIs within this population, using a before-and-after controlled trial design.

### Study Design

The study evolved from a 2024 local public health intervention pilot. During the ethical review in June 2024, the local ethics committee recommended that, given the sensitivity and vulnerability of university student participants, all eligible students should receive access to the intervention resources and that establishing a nonintervention control group would be ethically inappropriate. Considering this recommendation and the small, hard-to-reach population size, a randomized controlled trial was deemed infeasible. Therefore, a before-after self-controlled cohort design was adopted, allowing participants to serve as their own controls to assess within-person changes.

Guided by findings from systematic reviews indicating that a 12-month intervention period is optimal for achieving behavioral changes [14], this study will set a 12-month intervention duration, with an interim follow-up assessment at 6 months.

### Study Population

#### Inclusion Criteria

Participants will be eligible for inclusion if they meet all of the following criteria: (1) male, aged 18 years or older; (2) currently enrolled university student (including students pursuing technical diplomas or undergraduate degrees); (3) self-identified MSM, defined as having engaged in sexual behaviors with men, including mutual masturbation, oral sex, or anal intercourse; (4) confirmed HIV-negative status at baseline screening; and (5) willing and able to provide written informed consent to participate in the study.

#### Sample Size

To estimate the required sample size for a prevalent cohort under an exponential distribution assumption, we adopted the 2-sample Poisson rate comparison framework described by Shiue and Bain [15]. Detailed formulas and derivations are provided in Multimedia Appendix 1.

We assumed a baseline HIV incidence of 4 per 100 person-years (estimated from multiple MSM cohort studies in China [16-18]) and an expected postintervention incidence of 1 per 100 person-years (a 75% relative reduction), with a 2-sided α of .05, 80% power, and an anticipated 10% attrition over 12 months. Based on these assumptions, the required sample size is 484 participants.

The projected reduction to 1 per 100 person-years (75%) was empirically derived. Continuous surveillance of the MSM population in Chengdu observed a 60.9% year-over-year decline in new HIV infections between 2022‐2023 and 2023‐2024 (internal monitoring data available upon request). Building upon this trend, our multilevel intervention—integrating digital education, peer counseling, and PEP subsidies—is expected to produce further improvement. Preexposure prophylaxis (PrEP) combination-prevention intervention internationally has achieved 31.5% relative risk reduction among MSM [19], thus a 75% reduction was adopted as an empirically grounded upper-bound planning scenario.

We anticipated a 12-month retention rate of approximately 90% in this online cohort of student MSM. This assumption is informed by previous longitudinal studies among young men who have sex with men and gay/bisexual men, which have reported 12-month follow-up retention of 85% to 91% under structured follow-up procedures and supportive engagement strategies [20,21]. Given that our study uses multiple retention strategies (scheduled online follow-ups, advance notification of all incentive payments at enrollment, and close coordination through student volunteers), a 10% loss to follow-up was considered an ambitious but achievable design target. Sensitivity analyses at 25%, 50%, and 75% relative reductions and attrition rates of 10% to 20% were prespecified at the fixed N to assess feasibility and robustness (see Multimedia Appendix 2 for summary scenarios).

### Study Setting

This study is scheduled to commence in June 2025 and conclude in June 2026. The research process will consist of the following sequential phases: participant recruitment, HIV/hepatitis C virus (HCV)/hepatitis B surface antigen (HBsAg)/syphilis testing, baseline survey, intervention implementation, midterm survey, and final follow-up survey.

### Enrollment Procedure

Participant recruitment will be conducted exclusively through two internet-based channels to maximize efficiency and ensure privacy. The recruitment period is expected to last for 1 month. To ensure balanced workload distribution, each volunteer will have a preassigned recruitment quota. Once a volunteer has reached their quota, subsequent applicants will be redirected to other available volunteers. Given the estimated total sample size and the involvement of 10 student volunteers, it is calculated that each volunteer will be responsible for recruiting approximately 50 eligible student MSM participants.

#### Recruitment via WeChat

Recruitment information will be disseminated through a dedicated WeChat official account (WeChat is a Chinese multipurpose messaging, social media, and mobile payment app developed by Tencent Inc), which will publish targeted enrollment announcements clearly specifying the study’s inclusion criteria. These announcements will also provide the WeChat contact information of designated student volunteers. Interested individuals will be instructed to contact these volunteers directly via WeChat, where the volunteers will conduct eligibility screening and coordinate preenrollment HIV and STI testing.

#### Recruitment via Blued

Additional recruitment will be conducted through Blued, a popular location-based social networking application specifically designed for MSM, developed by BlueCity Holdings (Beijing, China). Student volunteers will create recruitment accounts on Blued and prioritize outreach efforts at pilot universities—institutions identified through surveillance data as having historically higher HIV prevalence rates and larger student MSM populations.

Leveraging Blued’s geolocation features, volunteers will target MSM individuals within their assigned university campuses. Given Blued’s explicitly MSM user base, recruitment through this channel is expected to contribute at least 50% of the total sample size. To ensure consistency in intervention delivery, all participants recruited through Blued will also be required to add the corresponding volunteer’s WeChat account, through which the subsequent online intervention activities will be administered.

### Incentives

Participants who complete the baseline and follow-up survey will receive a modest monetary incentive. This compensation strategy serves two primary purposes: (1) to acknowledge participants’ time and effort and (2) to foster participant retention by establishing a sense of commitment and accountability within the study.

Specifically, participants received RMB 20 (US $2.83) at baseline, RMB 20 (US $2.83) at the 6-month follow-up, and RMB 20 (US $2.83) at the final 12-month follow-up. The incentive amounts were reviewed and approved by the Ethics Committee of the Chengdu CDC (Approval No. 2024021). The total compensation has been carefully calibrated to ensure it remains modest and noncoercive while promoting continued engagement.

This incentive structure also incorporates behavioral-economics principles such as loss aversion, in which participants risk forfeiting future incentives if they withdraw prematurely. This approach encourages consistent participation without exerting undue influence.

### HIV-HCV-HBsAg-Syphilis Testing

#### Overview

Participants will be required to complete self-testing for HIV, HCV, HBsAg, and syphilis at baseline, as well as during both follow-up surveys. Only individuals who test negative for HIV at baseline will be eligible for enrollment in the cohort. All participants are tested at each follow-up using a four-in-one rapid diagnostic kit that simultaneously detects HIV, syphilis, HCV, and HBV. Any reactive screening result triggers referral for confirmatory testing in certified laboratories. If a participant tests positive for any infection marker, they are immediately referred to a certified medical institution for confirmatory diagnosis and treatment, in accordance with national guidelines.

It should be specifically noted that we have also taken into account the issue of the serological window periods. To minimize potential misclassification, we have incorporated several measures including repeat testing for recent exposures and comprehensive risk documentation.

#### Repeat Testing for Recent Exposures

Participants who report any high-risk sexual exposure within the 3 months preceding a visit are scheduled for repeat rapid testing after 2‐4 weeks.

#### Comprehensive Risk Documentation

Behavioral data from the past 3 months are collected at each follow-up survey. Participants reporting recent high-risk exposure but negative results are flagged as “possible window-period” cases and analyzed in sensitivity analyses.

Peer volunteers continuously follow up with referred participants to document their confirmatory results. The same referral and verification process applies to HIV, HCV, and HBV. This workflow ensures that all reported cases represent laboratory-confirmed new infections, minimizing classification bias.

### HIV-HCV-HBsAg-Syphilis Self-Testing Procedure

#### Overview

HIV-HCV-HBsAg-Syphilis self-testing will be conducted using Rong Ai Jian, a WeChat-based point-of-care testing mini-program developed by the Chengdu CDC. This platform integrates CBOs engaged in HIV prevention efforts throughout Chengdu. Participants can request self-testing kits through the Rong Ai Jian mini-program on WeChat. The CBOs will mail the test kits directly to applicants, who will then complete self-testing, upload their results, and receive professional review and feedback via the platform. This mechanism has been operational for over 2 years and has demonstrated stable functionality and effectiveness in practice.

The platform provides a complete traceable workflow, ensuring the accuracy and authenticity of self-test results.

#### Kit Distribution and Coding

Each four-in-one rapid test kit is prelabeled with a unique ID number by the CBO staff before shipment.

#### Result Reporting

After completing the self-test according to the instruction sheet, participants are required to upload a clear photo of the used test kit through the Rong Ai Jian WeChat mini-program.

#### Real-time Verification

CBO staff can view the uploaded image immediately on the platform and verify the result visually. The staff will then record the verified result in the platform system by selecting “Negative,” “To Be Retested,” or “Invalid.”

#### Participant Confirmation

The verified result automatically appears in the participant’s mini-program interface. Participants can screenshot this verification and send it to their peer volunteer as proof of testing completion.

#### Referral for Confirmation

Cases marked as “To Be Retested” are treated as preliminary reactive results. Both the CBO staff and peer volunteer then refer the participant to a qualified medical institution for confirmatory testing.

#### Final Confirmation and Data Entry

Only results that are confirmed by certified laboratories (eg, by treponemal and non-treponemal tests for syphilis or confirmatory assays for HIV/HBV/HCV) are entered as positive outcomes in the research database.

### HIV-HCV-HBsAg-Syphilis Combo Rapid Testing

The study will utilize the quadruple rapid diagnostic test kit developed by Guangzhou Wondfo Biotech Co., Ltd. This assay enables the simultaneous qualitative detection of HIV-1/2 antibodies, HCV antibody, HBsAg, and Treponema pallidum antibody from whole blood, serum, or plasma samples. The test employs a lateral flow immunochromatographic assay and provides results within 15 minutes.

Each test kit includes a test cassette, desiccant pouch, dropper, and buffer vial. The HIV-HCV-HBsAg-Syphilis Combo Test is approved by the China National Medical Products Administration, ensuring its safety and efficacy for clinical application.

Independent evaluations have reported excellent diagnostic performance, with sensitivities of 99.70% for HIV, 98.71% for HCV, 98.97% for HBsAg, and 98.47% for syphilis, along with corresponding specificities of 100%, 99.33%, 99.78%, and 99.68%, respectively. This highly reliable and user-friendly assay is particularly well-suited for point-of-care testing and large-scale screening initiatives, enabling timely diagnosis and facilitating rapid linkage to appropriate health care services [22].

### Questionnaire Survey

Baseline data collection will be conducted via an online self-administered questionnaire. Assigned project volunteers will securely deliver the survey link to participants through encrypted messaging channels.

### Electronic Informed Consent

An electronic informed consent process will be embedded at the beginning of the baseline questionnaire. Participants will be required to carefully read the informed consent form, which details the purpose of the study, potential risks and benefits, data confidentiality measures, and their rights as participants. Only after acknowledging their understanding and agreement will participants be permitted to proceed with completing the survey.

### Generation of Unique Participant ID and Data Security

To ensure participant anonymity while allowing for longitudinal data linkage, a de-identified participant code will be generated for each individual at enrollment. The participant ID consists of a randomly generated alphanumeric string created through an independent encryption algorithm, ensuring that no elements of phone numbers, timestamps, or other personally identifiable information (PII) are embedded in the code. The mapping file linking IDs with PII will be stored separately in an encrypted database accessible only to authorized Chengdu CDC data managers.

All study data are managed within a comprehensive data protection and security framework, consistent with national and international privacy standards. PII (such as phone numbers, WeChat IDs, and mailing addresses) is collected solely for operational purposes and stored separately from deidentified research data. Both databases are encrypted at rest and during transmission using the Advanced Encryption Standard (AES-256) encryption protocol implemented within the Chengdu CDC secure data environment.

A Data Protection Impact Assessment was performed to identify potential privacy risks and define mitigation strategies, including data-flow mapping, encryption and storage measures, access-control policies, retention and deletion schedules, and oversight procedures.

Access to data is governed by role-based access control, ensuring that each staff member only accesses the data required for their function. Multifactor authentication (eg, password+phone verification) is required for system login. All access and data modification activities are recorded through automated audit logs, which are periodically reviewed by the Chengdu CDC data security officer.

In accordance with the approved data retention and deletion schedule, PII will be retained for up to 6 months after study completion and then permanently deleted from all servers. Deidentified research data will be securely stored for 5 years for potential secondary analysis and subsequently removed after encrypted archiving.

These combined administrative, technical, and procedural safeguards ensure participant anonymity and protect the confidentiality, integrity, and traceability of all data throughout the study lifecycle.

### Survey Content and Follow-Up

Participants will complete a baseline questionnaire upon enrollment, which collects detailed information on sociodemographic characteristics, sexual behaviors, HIV-related knowledge, attitudes, practices, and other relevant behavioral factors (see Multimedia Appendix 3 for details).

Follow-up surveys will be administered at two time points post-intervention: 6 months and 12 months. The content of the follow-up questionnaires will be identical to that of the baseline survey to enable assessment of changes over time (see Multimedia Appendix 4 for follow-up questionnaire content). Data collection instruments and operational manuals have been standardized to ensure consistency in measurement and facilitate reproducibility across different investigators.

### Intervention Process

#### Developing Intervention Package

**Figure 1.**
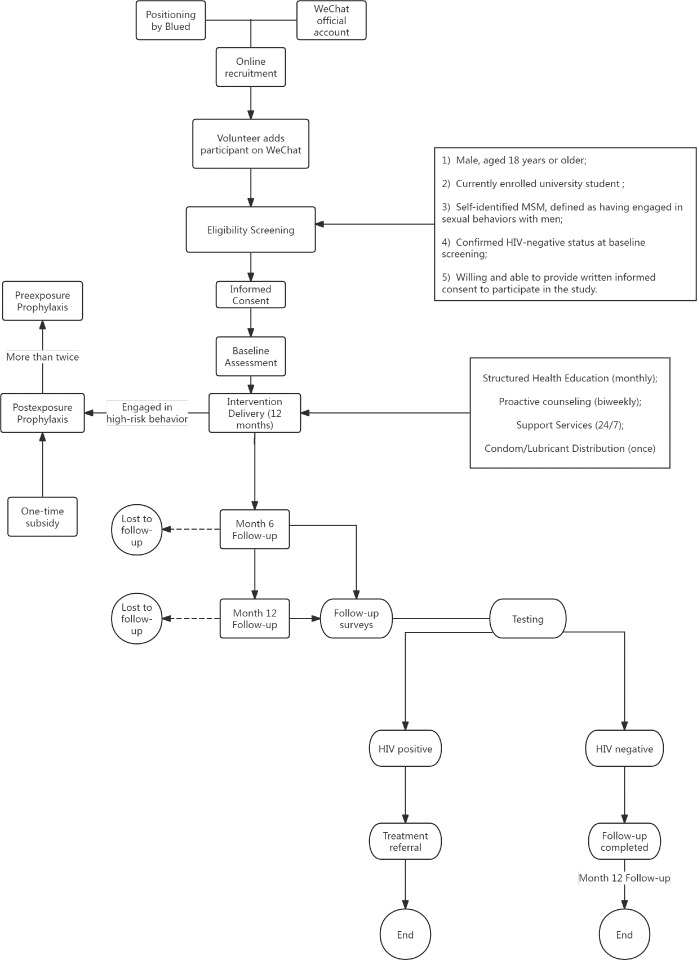
Participant flow and intervention process of the study. Rounded rectangles denote the core procedural stages of participant enrollment and intervention and follow-up. Stadium-shaped nodes indicate critical follow-up checkpoints. Circles represent study end points, including completion and loss to follow-up. Square boxes indicate detailed information units (eg, eligibility criteria and intervention components). Solid arrows indicate participant progression through the study procedures, while dashed arrows indicate attrition paths. MSM: men who have sex with men.

The intervention will be conducted entirely online over a 12-month period. Under the guidance of HIV prevention experts and CBO trainers, we have developed a comprehensive and targeted intervention package for participants. This package includes a standardized online intervention workflow (Figure 1), evidence-based web-based content, and accessible links to self-testing kits and referral services. A detailed schematic of the participant flow and multilevel intervention process is presented in Figure 1. The diagram depicts the sequential stages of recruitment, screening, enrollment, baseline assessment, intervention delivery, and follow-up. Detailed standard operating procedures have been developed to standardize the delivery frequency, message content, and volunteer-participant interactions, ensuring consistency across all intervention components. To ensure consistency and standardization in the delivery of peer-led interventions, the project implements a multilayered training, certification, and supervision system.

#### Standardized Training Curriculum

All peer volunteers undergo a unified, structured training program jointly conducted by the Chengdu CDC and partner CBOs. The training covers HIV prevention knowledge, motivational interviewing skills, online communication protocols, and ethical considerations (confidentiality and noncoercive engagement).

#### Posttraining Assessment and Certification

After training, each volunteer must pass a competency assessment that evaluates communication, adherence to intervention procedures, and knowledge of referral pathways. Only volunteers who pass this assessment are certified to conduct interventions.

#### Guideline Support

Each volunteer receives a “Guidelines for Internet-Based HIV Interventions,” which includes frequently asked questions and recommended responses. This guideline serves as a reference to ensure uniform content delivery and quality control during intervention sessions.

#### On-Site and Ongoing Supervision

Experienced CBO staff serve as trainers and provide continuous mentorship to peer volunteers throughout the intervention phase. These trainers, together with CDC supervisors, monitor intervention implementation through regular check-ins, feedback sessions, and review of anonymized communication logs when needed.

### Capacity Building for Project Volunteers

To enhance the understanding of the student MSM volunteers on the project rationale and improve their capacity to implement interventions, a structured training program will be provided prior to project initiation. This training will include both theoretical and practical components, such as (1) lectures to be delivered by experts and CBO trainers on HIV prevention strategies (eg, PrEP and PEP), online counseling skills, and psychological communication techniques; (2) practical simulation exercises focusing on real-world scenarios and case discussions; (3) orientation sessions explaining project-specific tasks, performance assessment indicators, and operational guidelines.

### Intervention Implementation

#### Overview

Trained student volunteers will deliver the intervention package to participants via Enterprise WeChat (a secure communication platform). The package will consist of four key components: structured health education modules, proactive counseling services, PEP assistance, and HIV prevention supply distribution.

#### Structured Health Education Modules

Monthly educational content will be disseminated, featuring curated articles from authoritative HIV prevention platforms operated by CBOs or national organizations (eg, China CDC official accounts). Each module focuses on a specific theme; content development will prioritize cultural relevance and adolescent-friendly design, utilizing infographic-style visual prescriptions tailored to student MSM cognitive preferences.

Educational content will adhere to national guidelines, including Guidelines for HIV Prevention among MSM [23] and Guidelines for Internet-based HIV Interventions [24]. Core content areas will include (1) HIV/STI transmission risks and condom-compatible lubricant usage; (2) PEP/PrEP knowledge and access pathways (eg, initiation within 72 h for PEP); (3) geolocation-enabled testing site information and MSM-specific epidemiological updates; and (4) risks associated with the use of addictive sexual enhancement products.

#### Proactive Counseling Services

Each participant receives biweekly proactive one-on-one counseling conducted by peer volunteers using motivational-interviewing techniques to reinforce safer sexual behaviors and strengthen adherence to preventive measures. These online sessions typically last 10 to 15 minutes and are delivered through WeChat. Core topics include HIV risk-reduction planning, emotional well-being, stigma coping, and linkage to HIV testing or care services.

In addition, a 24/7 online crisis-support channel is available for participants experiencing psychological distress, substance-use crises, or urgent HIV-related concerns. Volunteers are trained to provide first-line counseling and escalate complex cases to CDC supervisors or professional counselors within 24 h.

#### PEP Assistance

For participants reporting high-risk exposures (eg, condomless anal intercourse or needle-sharing), volunteers will provide the following:

Rapid linkage to PEP services: Same-day appointment coordination with PEP-prescribing outpatient clinics using a geolocation database.

Financial assistance: A one-time subsidy of RMB 500 (US $70.88) will be provided upon verified PEP completion (submission of clinic-issued proof). All PEP referrals are monitored through CDC-verified reporting channels to ensure timely initiation and adherence support. Participants with repeated high-risk exposures were assessed for PrEP eligibility in accordance with the Expert Consensus on HIV PrEP in China [25].

#### HIV Prevention Supply Distribution

Participants will receive a discreetly packaged prevention kit via third-party logistics, ensuring privacy protection. The kit will include condoms, water-based lubricants, and STI self-testing kits.

Participants will receive a discreetly packaged prevention kit via third-party logistics, ensuring privacy protection. The kit will include condoms, water-based lubricants, and STI self-testing kits.

Distribution will be limited to a one-time delivery per participant to optimize resource allocation.

Volunteers track delivery confirmation through the digital management platform.

### Study End Points

The study has three predefined endpoints:

1. HIV seroconversion: Participants with a reactive HIV test result will receive posttest counseling and referral to the local CDC for confirmatory laboratory testing. If HIV infection is laboratory-confirmed, the participant will be withdrawn from further study follow-up and intervention activities.
2. Loss to follow-up: Participants will be considered lost to follow-up if they fail to complete follow-up assessments and cannot be contacted despite repeated attempts.
3. Study completion: Participants who complete the 12-month follow-up period and final HIV/STI testing will be considered as having completed the study.

### Outcome Measures

#### Primary Outcome

HIV and syphilis incidence rate: The primary outcome is the change in HIV and syphilis incidence rates before and after the intervention, assessed by comparing the postintervention incidence with the baseline incidence among local student MSM populations.

#### Secondary Outcomes

Secondary outcome measures include (1) changes in sexual behaviors (eg, condom use, number of sexual partners) before and after the intervention; (2) frequency of biomedical intervention utilization (eg, HIV testing, STI testing, PEP, PrEP use); and (3) changes in HIV and STI prevention knowledge and awareness levels.

Detailed definitions of outcome indicators, measurement methods, and data sources are summarized in Table 1.

**Table 1.** Indicators of study outcomes.

Domain	Specific indicator	Definition	Data source
Primary outcomes			
HIV incidence	Comparison between intervention cohort and local student MSM attending university	New HIV cases confirmed by medical institutions per 100 person-years vs surveillance data (2025)	Laboratory reports at 12-month follow-up; CDC^b^ surveillance data
Syphilis incidence	Comparison between intervention cohort and local student MSM attending university	New syphilis cases confirmed by medical institutions per 100 person-years vs surveillance data (2025)	Laboratory reports at 12-month follow-up; CDC surveillance data
Secondary outcomes			
Behavioral Changes			
Risk behaviors	Condom use during anal intercourse Commercial sex engagement Multiple partners	% reporting “always use condoms” in past 3 months % reporting paid sex in past 3 months % with ≥2 sexual partners in past 3 months	Structured questionnaire
Substance use	Psychoactive substance use during sex	% reporting use of chemsex drugs in past 3 months	Structured questionnaire
Testing behaviors	HIV testing frequency STI^c^ screening	% tested for HIV in past 3 months % tested for STIs in past 3 months	Structured questionnaire
Biomedical uptake			
Prevention tools	Condom/lubricant use PrEP^d^/PEP^e^ utilization	% using condom/lubricant % PrEP/PEP initiations	Supply logs; pharmacy data
Attitudinal changes			
Prevention intention	Future condom use intent Testing willingness	% planning to use condoms % willing to undergo future HIV/STI tests	Structured questionnaire
Awareness changes			
Health literacy	PEP/PrEP knowledge HIV/STIs awareness	% correctly describing PEP/PrEP protocols % choosing the right core knowledge about HIV/STIs	Knowledge assessment

a MSM: men who have sex with men.

b CDC: Centers for Disease Control and Prevention.

c STI: sexually transmitted infection.

d PrEP: preexposure prophylaxis.

e PEP: postexposure prophylaxis.

### Participant Timeline

Time schedule of enrollment, interventions, assessments, and visits for participants are presented in Table 2.

**Table 2.** Schematic diagram for the schedule of enrollment, interventions, and assessments.

	Study period								
Timepoint and enrollment	Allocation	Postallocation							Close-out
	M0^a^	M1	M2	M3	M4	M5	M6	M7-11	M12
Enrollment									
Recruitment	✓								
Eligibility screen	✓								
Informed consent	✓								
Intervention									
Health education		✓	✓	✓	✓	✓	✓	✓	
Counseling services		✓	✓	✓	✓	✓	✓	✓	
HIV prevention supply distribution		✓							
PEP^b^ assistance		✓	✓	✓	✓	✓	✓	✓	
Assessments									
Baseline survey	✓								
Follow-up survey							✓		✓
Testing							✓		✓

a M: Month

b PEP: postexposure prophylaxis.

### Data Management

Survey data will be collected through Wenjuanxing, an online survey platform developed and operated by Ranxing Information Technology Co., Ltd., based in Changsha, China [26]. Wenjuanxing provides an automated data archiving system designed to ensure the integrity, security, and consistency of submitted responses.

All survey data will be stored on secure servers hosted by Alibaba Cloud in Hangzhou, China. The server infrastructure is protected by enterprise-level firewall systems, with daily automated data backups to prevent potential data loss and ensure disaster recovery capabilities.

Access to the database is strictly controlled through a role-based access control system. Only authorized research personnel, who have signed confidentiality agreements, will be granted access to view or manage the data. User authentication protocols, including secure passwords and encrypted data transmission, are implemented to further enhance data security and prevent unauthorized access.

### Statistical Analysis

Upon completion of data collection, all survey data will be exported from the Wenjuanxing platform for data cleaning and preprocessing to ensure accuracy, completeness, and consistency. Data management and analysis will be conducted using established statistical software packages, including SPSS, Stata, or R.

Descriptive statistics will be used to summarize participant characteristics and key study variables. Categorical variables will be presented as frequencies and percentages, while continuous variables will be reported as means with SD or medians with IQR, depending on data distribution. The normality of continuous variables will be assessed using Shapiro-Wilk tests and visual inspection of histograms/Q–Q plots. Univariate analyses, such as chi-square tests for categorical variables and *t* tests or nonparametric tests for continuous variables, will be used to assess preliminary associations between independent variables and outcome measures. Multivariate analysis will be performed using logistic regression models (for binary outcomes) to identify factors independently associated with key study end points, adjusting for potential confounders. If advanced statistical modeling (eg, Cox proportional hazards models, generalized estimating equations) is deemed necessary to address specific research questions or to handle longitudinal data, additional statistical expertise will be sought to ensure appropriate application and interpretation of these methods. A 2-sided *P* value <.05 will be considered statistically significant.

### Feasibility and Acceptability Evaluation

Feasibility will be assessed by examining recruitment and retention rates, completeness of follow-up surveys, and timeliness of testing. Acceptability will be evaluated through participant satisfaction surveys and qualitative feedback collected from peer volunteers regarding the practicality and perceived usefulness of the intervention materials. Descriptive statistics will be used to summarize feasibility and acceptability outcomes, and qualitative comments will be thematically analyzed.

## Results

Participant enrollment and data collection are currently ongoing. This trial protocol has been developed in accordance with the SPIRIT (Standard Protocol Items: Recommendations for Interventional Trials) 2013 guidelines [27].

As of May 1, 2025, the intervention materials and training program have been finalized, and recruitment has begun. Enrollment of participants started in April 2025. By the end of April, 127 participants had completed baseline surveys and HIV/STI self-tests.

## Discussion

### Key Innovation Points

This prospective cohort study will be uniquely designed to evaluate the effectiveness of an internet-based intervention in reducing HIV-related high-risk behaviors and the prevalence of HIV and other STIs among university student MSM. Beyond addressing the primary study objectives, the research will innovatively integrate digital engagement strategies with peer-led support, representing a pioneering approach within this population.

Specifically, the study will establish a dedicated intervention cohort that will utilize social networking platforms to deliver continuous behavioral interventions, promote HIV/STI testing, facilitate service referrals, and provide financial subsidies to support access to PEP.

A key innovation of this study will be the application of the Network Intervention Cohort Peer Model, which will involve recruiting experienced staff from MSM CBOs as trainers and engaging university student MSM volunteers as frontline interventionists. This model is expected to enhance the cultural relevance and acceptability of the intervention while addressing critical limitations of conventional peer education programs.

Furthermore, all interventions will be delivered entirely online, aligning with current trends in MSM-focused health promotion. The intervention package will integrate both behavioral and biomedical components—including the discreet distribution of condoms and lubricants by mail, as well as financial subsidies to support timely initiation of PEP when indicated. Prior research has demonstrated that eliminating financial barriers is a key strategy for empowering MSM to access and sustain engagement with essential HIV prevention services [28].

Recent regulatory tightening surrounding CBO activities—particularly in relation to direct engagement with university students—has significantly constrained the scope of traditional peer education efforts. While Chengdu has previously mobilized college student volunteers through partnerships with local CDCs and AIDS Prevention Associations, such initiatives have generally been broad-based and not specifically tailored to the distinct needs of university student MSM.

Against this backdrop, our project will represent the first known effort in China to design and implement a comprehensive, peer-led network intervention model specifically targeting this demographic.

Despite the study’s innovative strengths, several anticipated operational challenges merit careful consideration.

### Standardization of Volunteer Training and Mentoring

The project relies on CBO staff serving as trainers and student MSM volunteers acting as interventionists. Ensuring consistency and quality in training and mentoring is critical to maintaining intervention fidelity. Variability in training delivery or gaps in knowledge may lead to inconsistent intervention outcomes.

To address this, trainers will be selected from experienced CBO staff across Chengdu with a demonstrated track record in intervention delivery and volunteer mentoring. All volunteers are required to complete the entire capacity-building training and pass a posttraining assessment before participating in interventions. During the intervention process, volunteers can consult their trainers at any time regarding operational issues, such as making referrals or responding to participant inquiries, and can also seek support through the project’s online group chat.

### Recruitment and Retention of a Fixed Cohort

Maintaining participant retention throughout the 12-month follow-up period is crucial to ensuring sufficient statistical power and minimizing attrition bias. While multiple recruitment strategies and financial incentives have been planned, challenges in achieving the targeted sample size may still arise.

To mitigate this risk, contingency plans include expanding recruitment channels, such as peer referrals and targeted social media advertisements, if enrollment falls short. Additionally, each participant will be required to provide both their WeChat contact and mobile phone number upon enrollment. Participants will also be informed that this information, along with their mailing address, will be used solely for study-related purposes, such as the confidential delivery of condoms, lubricants, and other prevention materials, which may help improve retention and engagement.

### Data Collection and Baseline Assessment: Ensuring Reliability of Self-Reported Data

The baseline survey collects sensitive data, including sexual behaviors, substance use, and HIV-related risk factors. Due to social desirability bias or privacy concerns, participants may underreport or misreport behaviors, potentially affecting data accuracy.

To address this, volunteers will emphasize the project’s strict confidentiality protocols when inviting participants to complete the questionnaire. Participants will be clearly informed that no PII is collected in the survey, all data are securely stored in the back-end system, and only the project manager has access to detailed records.

This study has limitations. The before-after self-controlled cohort design does not include a concurrent control group, which limits the ability to make causal inferences. However, a randomized controlled trial was not feasible due to ethical and operational considerations—specifically, the local ethics committee’s recommendation that all eligible university students should have access to the intervention resources. To mitigate this limitation, we will compare changes observed in this study with contemporaneous surveillance data from the Chengdu student MSM population, conduct sensitivity analyses to assess secular trends, and interpret outcomes cautiously in light of these contextual factors. Despite these constraints, the design remains appropriate for evaluating real-world implementation and effectiveness under routine public health conditions. While the assumed reduction in incidence may be optimistic, behavioral indicators such as HIV testing and PEP/PrEP uptake serve as sensitive early markers of intervention impact in this short-term cohort. Given the expected rarity of incident infections over 12 months in student MSM cohorts, we interpret incidence changes cautiously while relying on proximal behavioral outcomes with higher short-term sensitivity.

By proactively addressing these anticipated challenges through standardized training, robust recruitment and retention strategies, and comprehensive privacy protections, this study aims to ensure rigorous implementation while generating valuable insights into the feasibility and effectiveness of internet-based, peer-led HIV prevention interventions among university student MSM.

## Supplementary material

10.2196/77078 Multimedia Appendix 1 Formulas and derivations.

10.2196/77078 Multimedia Appendix 2 Summary scenarios.

10.2196/77078 Multimedia Appendix 3 Baseline questionnaire.

10.2196/77078 Multimedia Appendix 4 Follow-up questionnaire.

## Abbreviations

CBO: community-based organization
CDC: Centers for Disease Control and Prevention
HBsAg: hepatitis B surface antigen
HCV: hepatitis C virus
MSM: men who have sex with men
PEP: postexposure prophylaxis
PII: personally identifiable information
PrEP: preexposure prophylaxis
SPIRIT: Standard Protocol Items: Recommendations for Interventional Trials
STI: sexually transmitted infection
