# Supplementary material for: A Multilevel Network Peer Intervention Among Student Men Who Have Sex With Men Attending University: Protocol for an Implementation-Effectiveness Before-After Cohort Study
Source: JMIR Res Protoc. 2026 Jan 23;15:e77078. doi: 10.2196/77078 (PMC12829898; doi:10.2196/77078)
Supplement: Multimedia Appendix 1 [file resprot-v15-e77078-s001.docx]

Appendix 1. Formulas and derivations.

The required number of pre-intervention person-years (λ_1_) is calculated as:

λ_1=_n_1_s_1_γ_1_($\sqrt{\frac{1+d\rho}{d+\rho}}$ $Z_{1-\alpha}+Z_{p}$)^2^/$(\rho-1)^{2}$

where:

n_1_:Estimated sample size for the pre-intervention groups

n_2_:Estimated sample size for the post-intervention groups

γ_1_:baseline incidence rate=0.04 person-years (estimated from multiple MSM cohort studies in China[17–19])

γ_2_:expected post-intervention incidence rate = 0.01 person-years

ρ:γ_2_/γ_1_=0.25,representing a 75% relative reduction

s_1=_s_2_=1 year

d=n_2_s_2_/n_1_s_1_=1(before–after self-controlled design)

α=0.05 (two-sided), z_α/2_=1.96.

p=0.8 (80% power), z_p_=0.84.

Substituting these values yields:

λ_1_ ≈17.42,n_1=_λ_1_/s_1_γ_1_≈436

Assuming 10% attrition, the final required sample size is:

n≈484

[17] *HIV incidence among men who have sex with men using geosocial networking smartphone application in beijing, China: An open cohort study | infectious diseases of poverty*. https://mednexus.org/doi/full/10.1186/s40249-021-00814-7 (accessed 2025-04-16).

[18] You X, Gilmour S, Cao W, Lau J T, Hao C, Gu J, et al. HIV Incidence and Sexual Behavioral Correlates among 4578 Men Who Have Sex with Men (MSM) in Chengdu, China: A Retrospective Cohort Study. *BMC Public Health* **2021**, *21* (1), 802. https://doi.org/10.1186/s12889-021-10835-4.

[19] Dai Z, Mi G, Yu F, Chen G, Wang X, He Q. Using a Geosocial Networking App to Investigate New HIV Infections and Related Risk Factors Among Student and Nonstudent Men Who Have Sex With Men in Chengdu, China: Open Cohort Study. *Journal of Medical Internet Research* **2023**, *25* (1), e43493. https://doi.org/10.2196/43493.
