# Supplementary material for: A Multilevel Network Peer Intervention Among Student Men Who Have Sex With Men Attending University: Protocol for an Implementation-Effectiveness Before-After Cohort Study
Source: JMIR Res Protoc. 2026 Jan 23;15:e77078. doi: 10.2196/77078 (PMC12829898; doi:10.2196/77078)
Supplement: Multimedia Appendix 2 [file resprot-v15-e77078-s002.docx]

Appendix 2. Summary scenarios.

Table S1. Sensitivity and feasibility scenarios for incidence detectability at fixed sample size (N = 484, 12-month follow-up)

| Scenario | Relative reduction (vs 4 / 100 PY) | Attrition (%) | Effective person-years (PY)* | Expected baseline events (4 / 100 PY) | Expected post-intervention events | Qualitative evaluability |
| --- | --- | --- | --- | --- | --- | --- |
| A | 75% (4 → 1 / 100 PY) | 10 | ≈ 459.8 | ≈ 18.4 | ≈ 4.6 | Marked difference; incidence reduction likely detectable with good power |
| B | 50% (4 → 2 / 100 PY) | 10 | ≈ 459.8 | ≈ 18.4 | ≈ 9.2 | Moderate difference; detection possible but with lower precision |
| C | 25% (4 → 3 / 100 PY) | 10 | ≈ 459.8 | ≈ 18.4 | ≈ 13.8 | Small difference; incidence change likely underpowered at α=0.05, 80% power |
| D | 75% (4 → 1 / 100 PY) | 20 | ≈ 435.6 | ≈ 17.4 | ≈ 4.4 | Detectable; slightly fewer events due to higher attrition, but still a clear contrast |
| E | 50% (4 → 2 / 100 PY) | 20 | ≈ 435.6 | ≈ 17.4 | ≈ 8.7 | Moderate difference; marginal power, broader confidence intervals expected |
| F | 25% (4 → 3 / 100 PY) | 20 | ≈ 435.6 | ≈ 17.4 | ≈ 13.1 | Limited evaluability; incidence difference difficult to detect with current N |
