# Supplementary material for: A Multilevel Network Peer Intervention Among Student Men Who Have Sex With Men Attending University: Protocol for an Implementation-Effectiveness Before-After Cohort Study
Source: JMIR Res Protoc. 2026 Jan 23;15:e77078. doi: 10.2196/77078 (PMC12829898; doi:10.2196/77078)
Supplement: Multimedia Appendix 3 [file resprot-v15-e77078-s003.docx]

Appendix 3: Baseline Questionnaire (English Version)

Informed Consent for Participation

Dear Participant,

You are invited to participate in a research project approved by the Chengdu Center for Disease Control and Prevention (CDC), which focuses on health intervention among university men who have sex with men (MSM). To protect your rights, this study has undergone ethical review and approval. This informed consent form introduces the study and outlines important information relevant to your participation. Please read it carefully and decide whether you are willing to participate.

The study aims to reduce new HIV and sexually transmitted infections (STIs) among MSM students through internet-based behavioral intervention. We will ask questions about your basic demographics, HIV knowledge, sexual behavior, testing, and biomedical prevention. Some questions may involve sensitive topics and may cause discomfort. All collected information will be used solely for HIV/STI prevention and will be kept strictly confidential by designated personnel. No personal identity information will be disclosed in any public report.

Your participation is completely voluntary. You may refuse to participate or withdraw at any point without any negative consequences for your current or future medical care. If you encounter any problems or have questions during the study, please contact local CDC staff. For ethical concerns, you may contact the Ethics Committee of Chengdu CDC at 028-87032247. The questionnaire will take approximately 5 minutes to complete.

Participant Consent Statement:

I have read and understood the information above, including potential risks and benefits. I voluntarily agree to participate in this study.

① Yes  ② No

Date of survey: ________ (YYYY/MM/DD)

Unique ID: __________ (generated automatically)

Section 1: Eligibility and Basic Information

Name of your university: ______________

Phone number: _____________________

Q1.Through which channel were you recruited into this intervention project?

 (1) Blued (2) WeChat article (3) Offline referral (please specify name): ________

 (4) Phone/SMS (5) Other: __________

Q2.Your age: ______ years

Q3.What is your biological sex?

 ① Male ② Female (End survey)

Q4.Are you currently a university student?

 ① Yes ② No (End survey)

Q5.Have you ever engaged in anal or oral sex with a male partner?

 ① Yes ② No (End survey)

Section 2: HIV Knowledge

C01. Is AIDS an incurable and serious infectious disease? ① Yes ② No ③ Don’t know

C02. Are MSM currently the most affected group by HIV in China? ① Yes ② No ③ Don’t know

C03. Can you tell if someone has HIV just by appearance? ① Yes ② No ③ Don’t know

C04. Does having another STI increase the risk of HIV infection? ① Yes ② No ③ Don’t know

C05. Does using new drugs (e.g., meth, ecstasy, ketamine) increase the risk of HIV infection?

 ① Yes ② No ③ Don’t know

C06. How would you rate your own risk of acquiring HIV?

 ① None ② Low ③ Moderate ④ High/Very high

Section 3: Sexual Behavior and Biomedical Prevention

Q6.Your sexual role:

 ① Bottom only ② Top only ③ Both

Q7.In the past 3 months, how often did you use condoms during anal sex with men?

 ① Never ② Sometimes ③ Always ④ No anal sex (Skip to Q11)

Q8.In the past 3 months, have you engaged in commercial sex with men? ① Yes ② No

Q9.In the past 3 months, how often did you use condoms during sex with women?

 ① Never ② Sometimes ③ Always ④ No heterosexual sex

Q10.In the past 3 months, have you used any of the following substances during sex?

 ① Bottom capsules ② G-spot fluid ③ Ketamine

 ④ Methamphetamine ⑤ Heroin ⑥ Ecstasy

 ⑦ Rush/Poppers ⑧ Other: _______ ⑨ None

Q11.Did you use a condom the last time you had anal sex with a male partner? ① Yes ② No

Q12.Do you plan to use condoms in your next anal sex encounter with a male partner?

 ① Yes ② No ③ Not sure

Q13.How many sexual partners have you had in the past 3 months? _________

Q14.After engaging in high-risk behavior, did you actively seek HIV testing or counseling?

 ① Yes ② No

Q15.Have you ever taken pre-exposure prophylaxis (PrEP)? ① Yes ② No (Skip to Q19)

Q16.If yes, how did you take PrEP?

 ① One pill per day

 ② Two pills 2–24 hours before sex, then one pill 24h and 48h after sex

 ③ Irregular use

Q17.Have you ever taken post-exposure prophylaxis (PEP) after high-risk sex? ① Yes ② No

Q18.Have you taken an HIV test in the past 3 months? ① Yes ② No

Q19.Have you taken an STI test in the past 3 months? ① Yes ② No

Q20.What was the result of your most recent HIV test?

 ① Never tested (Skip to Q24) ② Negative ③ Positive ④ Uncertain ⑤ Don’t know

Q21.Where did you last get tested for HIV?

 ① Hospital ② CDC ③ Rong Ai Jian self-test ④ Rong Ai Jian appointment

 ⑤ Other: __________

Q22.Are you willing to take an HIV test in the future? ① Yes ② No ③ Not sure

Q23.Are you willing to take an STI test in the future? ① Yes ② No ③ Not sure
