# Supplementary material for: A Multilevel Network Peer Intervention Among Student Men Who Have Sex With Men Attending University: Protocol for an Implementation-Effectiveness Before-After Cohort Study
Source: JMIR Res Protoc. 2026 Jan 23;15:e77078. doi: 10.2196/77078 (PMC12829898; doi:10.2196/77078)
Supplement: Multimedia Appendix 4 [file resprot-v15-e77078-s004.docx]

Appendix 4: Follow-up Questionnaire

Phone number: _______________________

Section 1: HIV Knowledge

C01. Is AIDS an incurable and serious infectious disease?

① Yes ② No ③ Don’t know

C02. Are MSM currently the group most severely affected by HIV in China?

① Yes ② No ③ Don’t know

C03. Can HIV infection be identified by a person’s appearance?

① Yes ② No ③ Don’t know

C04. Does having another STI increase the risk of HIV infection?

① Yes ② No ③ Don’t know

C05. Does the use of new drugs (e.g., methamphetamine, ecstasy, ketamine) increase the risk of HIV infection?

① Yes ② No ③ Don’t know

C06. How would you assess your current risk of HIV infection?

① None ② Low ③ Moderate ④ High or very high

Section 2: Sexual Behavior and Prevention Practices

Q6. Your sexual role:

① Bottom only ② Top only ③ Both

Q7. In the past 3 months, how often did you use condoms during anal sex with men?

① Never ② Sometimes ③ Always ④ No anal sex (Skip to Q8)

Q8. In the past 3 months, did you engage in commercial sex with men?

① Yes ② No

Q9. In the past 3 months, how often did you use condoms during sex with women?

① Never ② Sometimes ③ Always ④ No heterosexual sex

Q10. In the past 3 months, which of the following substances did you use during sex?

① Bottom capsules ② G-spot liquid ③ Ketamine

④ Methamphetamine ⑤ Heroin ⑥ Ecstasy

⑦ Rush/Poppers ⑧ Other: ________ ⑨ None

Q11. Did you use the condoms and lubricants provided by the project?

① Did not use (please specify why): ________

② Used them

③ Did not request (please specify why): ________

Q12. Did you use a condom the last time you had anal sex with a male partner?

① Yes ② No

Q13. Do you plan to use a condom the next time you have anal sex with a male partner?

① Yes ② No ③ Not sure

Q14. How many sexual partners have you had in the past 3 months? _______

Q15. After engaging in high-risk behavior, did you seek HIV testing or counseling?

① Yes ② No

Q16. Has your understanding of pre- and post-exposure prophylaxis (PrEP/PEP) improved as a result of participating in this project?

① Yes ② No

Q17. Have you ever taken pre-exposure prophylaxis (PrEP)?

① Yes ② No (Skip to Q19)

Q18. If yes, how did you take it?

① One pill daily

② Two pills 2–24 hours before sex, then one pill 24 and 48 hours afterward

③ Irregular use

Q19. Have you taken post-exposure prophylaxis (PEP) after high-risk behavior?

① Yes ② No

Section 3: HIV/STI Testing and Attitudes

Q20. Have you had an HIV test in the past 3 months?

① Yes ② No

Q21. Have you had an STI test in the past 3 months?

① Yes ② No

Q22. What was the result of your most recent HIV test?

① Never tested (Skip to Q24) ② Negative ③ Positive ④ Uncertain ⑤ Don’t know

Q23. Where did you get your most recent HIV test?

① Hospital ② CDC ③ Rong Ai Jian mini-program (self-test)

④ Rong Ai Jian mini-program (appointment) ⑤ Other: __________

Q24. Are you willing to take an HIV test in the future?

① Willing ② Unwilling ③ Not sure

Q25. Are you willing to take an STI test in the future?

① Willing ② Unwilling ③ Not sure

Section 4: Evaluation of the Project

Q26. Has your understanding of monkeypox improved as a result of participating in this project?

① Yes ② No

Q27. Do you think regular health promotion and HIV/STI interventions like this project are helpful?

① Yes ② No
